# Simultaneous Endovascular Abdominal Aortic Aneurysm Repair and Open Repair of Common Femoral Artery Aneurysm: Short Case Series and Current Review

**DOI:** 10.3390/jcm14227988

**Published:** 2025-11-11

**Authors:** Spyros Papadoulas, Melina Stathopoulou, Andreas Tsimpoukis, Chrysanthi Papageorgopoulou, Konstantinos Nikolakopoulos, Nikolaos Krinos, Aliki Skandali, Petros Zampakis, Petraq Mustaqe, Agron Dogjani, Francesk Mulita, Vasileios Leivaditis

**Affiliations:** 1 Department of Vascular Surgery, General University Hospital of Patras, University of Patras Medical School, Rio, 26504 Patras, Greece; melinastath@gmail.com (M.S.); tsimpoukis.and@gmail.com (A.T.); chrisanthi.papageorg@gmail.com (C.P.); konstantinosn@yahoo.com (K.N.); nickkrinos@yahoo.gr (N.K.); alsk280502@gmail.com (A.S.); 2 Department of Radiology, General University Hospital of Patras, University of Patras Medical School, Rio, 26504 Patras, Greece; pzampakis@gmail.com; 3 Department of Vascular Surgery, General Hospital of Vlore, 9401 Vlore, Albania; dr.petraqmustaqe@gmail.com; 4 Department of General Surgery, University of Medicine, 1005 Tirana, Albania; agrondogjani@yahoo.com; 5 Department of General Surgery, General University Hospital of Patras, University of Patras Medical School, Rio, 26504 Patras, Greece; 6 Department of Cardiothoracic and Vascular Surgery, Westpfalz Klinikum, 67655 Kaiserslautern, Germany; vnleivaditis@gmail.com

**Keywords:** endovascular abdominal aorta, femoral artery aneurysm, synchronous repair, open repair

## Abstract

**Background:** Common femoral artery aneurysms are rare and are usually associated with aneurysms at other sites, mainly the aorta, iliac, popliteal, superficial femoral, and profunda femoral artery. This combination poses the challenge of synchronous repair for clinically relevant aneurysms. Although endovascular abdominal aortic aneurysm repair is the main type of treatment for abdominal aortic aneurysms nowadays, this is not true for common femoral aneurysms, where open repair remains the gold standard. These two distinct operations could be combined in a one-stage procedure when aortoiliac and common femoral aneurysms present simultaneously. This approach potentially saves time and costs, without increasing complications. **Methods:** A retrospective search was conducted in the Vascular Surgery Department database of a tertiary referral center for vascular surgery, covering procedures from January 2005 to May 2025. Patients were included if they had undergone simultaneous endovascular abdominal aortic aneurysm repair and open repair of a common femoral artery aneurysm. Clinical records, operative details, imaging studies, and follow-up data were reviewed. We additionally provide a literature review regarding this approach. This review additionally incorporates the current knowledge regarding the treatment of common femoral artery aneurysms. **Results:** Out of 668 endovascular abdominal aortic aneurysm repair procedures, three patients (0.45%) were identified. These three patients were among five patients who were treated for true common femoral artery aneurysm by open repair in the same time interval. All of the patients are currently in good condition without late complications. One patient, who had not performed any follow-up imaging, was diagnosed with large aneurysms at other sites, 10 years later. **Conclusions:** The combined one-stage endovascular abdominal aortic repair and open repair of a common femoral artery aneurysm by interposition grafting is technically a simple approach that led to satisfactory outcomes.

## 1. Introduction

Most peripheral arterial aneurysms in the femoral, popliteal, and tibial arteries are currently either degenerative aneurysms or posttraumatic pseudoaneurysms after catheterization, instrumentation, or previous revascularization procedures [1,2,3,4,5]. True femoral artery aneurysms (FAAs) after trauma have also been reported [6]. Rarely, connective tissue disorders, infective vasculitis, or genetic predisposition may be implicated [7,8,9,10,11]. They have also been detected in Behcet disease, Marfan syndrome, and arteriomegaly [6,12,13]. Reports of a common femoral artery aneurysm (CFAA) in the context of angiosarcoma are extremely rare [14,15].

Occasionally, simultaneous detection of aneurysms at other sites may take place [16,17]. If a CFAA is diagnosed, there is an 85% probability for an abdominal aortic aneurysm (AAA) to coexist [18]. On the contrary, a patient with an AAA has a 14% probability of having a concomitant aneurysm of the common femoral (CFA) or popliteal artery (PA) [18,19,20,21]. This coexistence is due to common risk factors and similar biochemical factors involved in the pathophysiology of these entities [22].

In these cases, if the aneurysms are symptomatic or have reached the diameter threshold for repair, the issue of simultaneous management arises. Endovascular abdominal aortic aneurysm repair (EVAR) is the main treatment strategy of AAAs nowadays [23,24]. On the other hand, endovascular repair (ER) for CFAAs is not widely used due to the anatomic characteristics of these aneurysms, as the bifurcation does not usually permit safe landing. The extreme mobility of the groin is another restrictive factor, and concerns about possible stent fractures or dislodgement in this location may arise [1,25,26]. Consequently, EVAR combined with open repair of the CFAA is a prudent approach in most cases [27]. Herein, we present a series of three patients who underwent simultaneous elective treatment, including EVAR combined with open repair of the CFAA by interposition grafting. This is a simple technique, and all patients had satisfactory outcomes. A current literature review is also presented, which additionally includes current concepts regarding CFFA treatment.

## 2. Materials and Methods

The study was conducted in conformity with ethical standards and guidelines. Ethical approval for the research was obtained from the institutional review board of the University Hospital of Patras (IRB no. 29/11-07-2025, approval date: 11 July 2025). The study adhered to the principles outlined in the Declaration of Helsinki regarding research involving human subjects. Written informed consent was obtained from the patients to use their clinical data after the ethics committee approval.

We conducted a comprehensive and systematic search of the medical database of the Department of Vascular Surgery, General University Hospital of Patras, Patras Medical School. It includes diagnostic codes, operative reports, radiologic imaging, and information for all surgical procedures. The search period extended from 1 January 2005 up to 31 May 2025. The primary search keywords employed were “endovascular abdominal aortic repair” and “femoral aneurysm.” Patients were included if they met the following criteria: (i) referring for vascular reconstruction; (ii) undergoing simultaneous EVAR and CFAA open repair; (iii) complete clinical and radiological data; (iv) available follow-up.

## 3. Results

**Figure 1:**
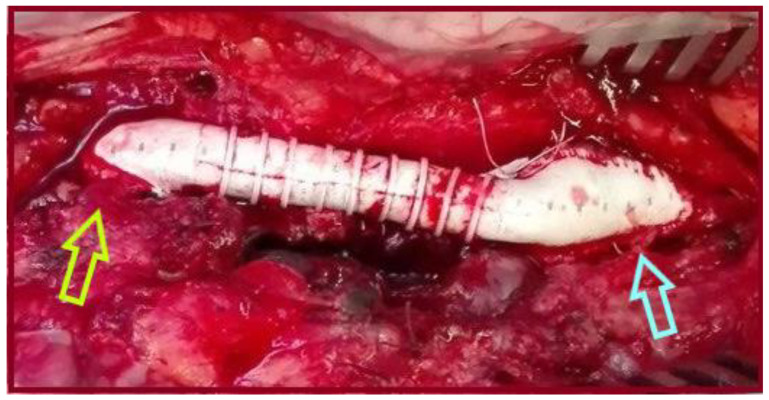
An 8mm-Ringed Polytetrafluoroethylene (PTFE) tube graft was interposed between the proximal common femoral artery and the common femoral bifurcation (light green arrow: proximal anastomosis, light blue arrow: distal anastomosis), to treat an isolated CFAA.

Out of 668 EVAR procedures performed in the searched period, we identified 3patients who met these criteria (0.45%), all of whom underwent successful operations. All cases were elective. Individual characteristics of the retrieved cases are reported below and summarized in Table 1. These three patients were among five patients who were treated for true CFAAs (one ruptured, one thrombosed)by open repair in the same time interval (Figure 1) [28]. Moreover, open repair was used to treat five superficial femoral artery aneurysms (SFAAs) (two ruptured) in this period.

### 3.1. Case 1

**Figure 2:**
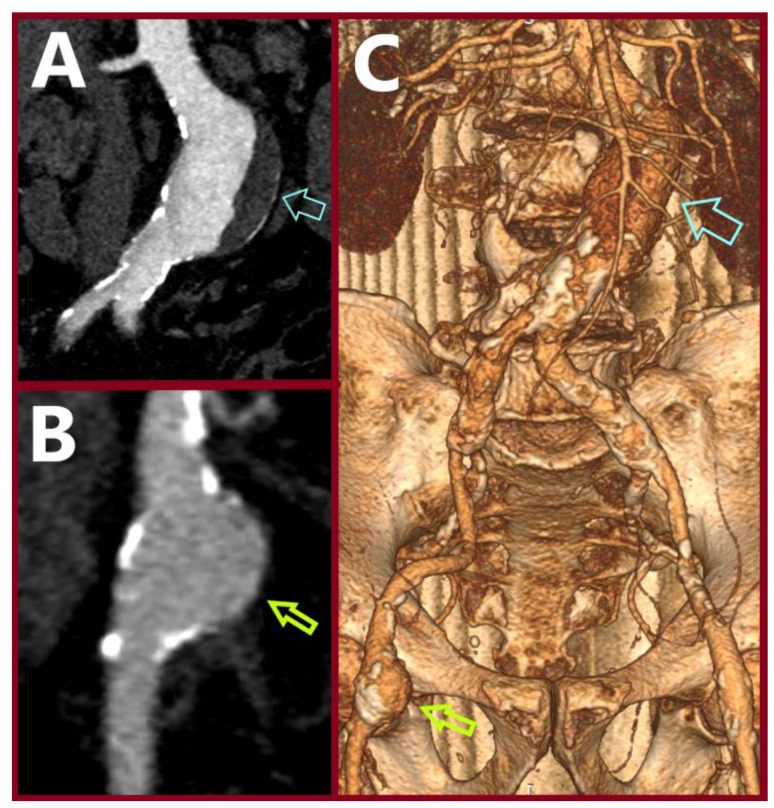
Preoperative Computed Tomography Angiography (CTA): (**A**) the abdominal aortic aneurysm (light blue arrow); (**B**) the common femoral artery aneurysm (light green arrow); (**C**) 3-D reconstruction depicting the abdominal (blue arrow) and the common femoral artery (green arrow) aneurysms.

**Figure 3:**
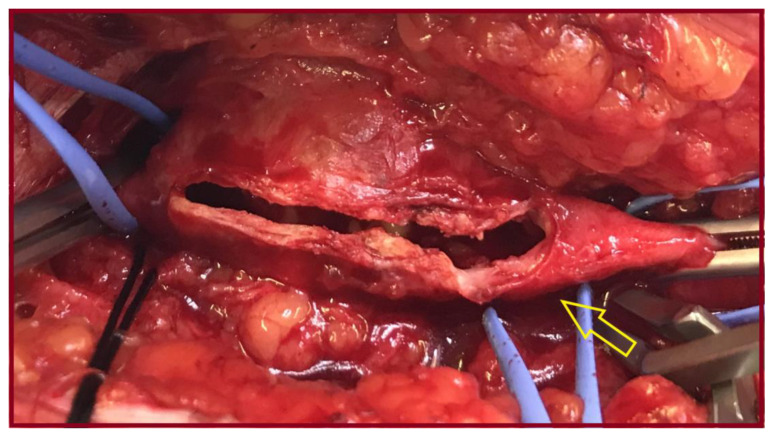
Intraoperative photo of the common femoral artery aneurysm, after opening the sac (yellow arrow: common femoral artery bifurcation).

**Figure 4:**
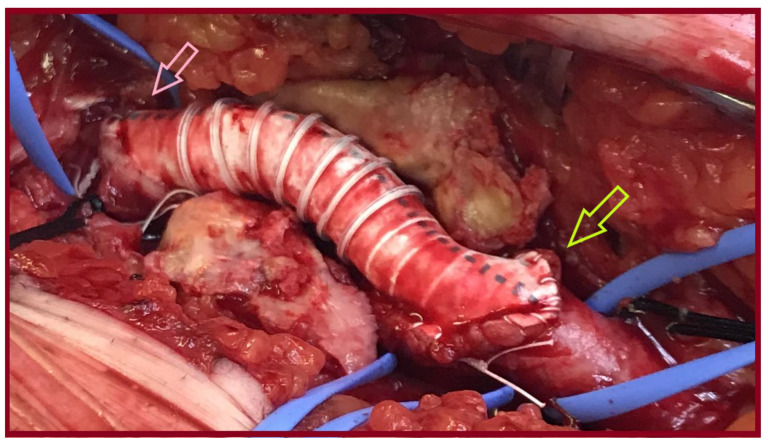
An 8mm-Ringed PTFE tube graft was interposed between the proximal common femoral artery and the common femoral bifurcation (pink arrow: proximal anastomosis, light green arrow: distal anastomosis).

A 76-year-old male presented with an asymptomatic AAA and a right CFAA sized 5.5 cm and 2.5 cm in diameter, on Computed Tomography Angiography (CTA), respectively (Figure 2). He was an ex-smoker, and his past medical history included atrial fibrillation. He received metoprolol tartrate 50 mg b.i.d. and apixaban 5 b.i.d. Operative management included a one-stage approach under general anesthesia. After femoral cutdown, the right CFAA was dissected and looped at the neck and the CFA bifurcation. A standard EVAR was accomplished with a Gore Excluder Endoprosthesis (W. L. Gore & Associates Inc., Flagstaff, AZ, USA). Vascular access was gained through the origin of the superficial femoral artery (SFA). Consequently, a standard CFAA repair with the inlay technique was performed using an 8mm Ringed e-PTFE graft (W. L. Gore & Associates Inc., Flagstaff, AZ, USA) (Figure 3 and Figure 4). Postoperative outcome was uneventful. He was discharged on the third postoperative (p.o.) day. A pacemaker was inserted 2 months later. During the follow-up period, abdominal imaging was normal. He died 5 years later from urinary bladder cancer.

### 3.2. Case 2

**Figure 5:**
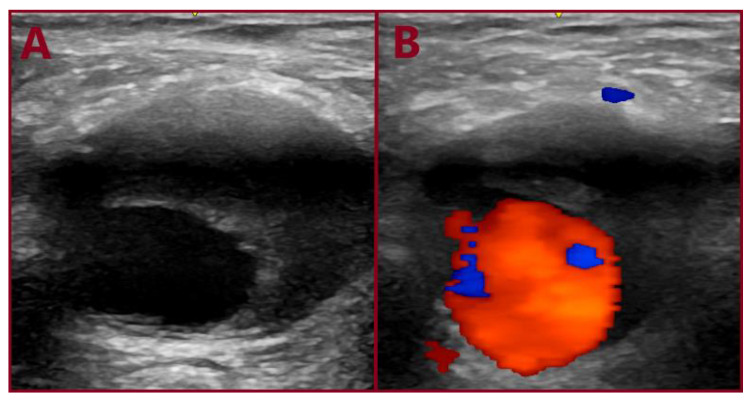
Ultrasonographic appearance of the common femoral artery aneurysm: (**A**) gray-scale; (**B**) color duplex imaging.

**Figure 6:**
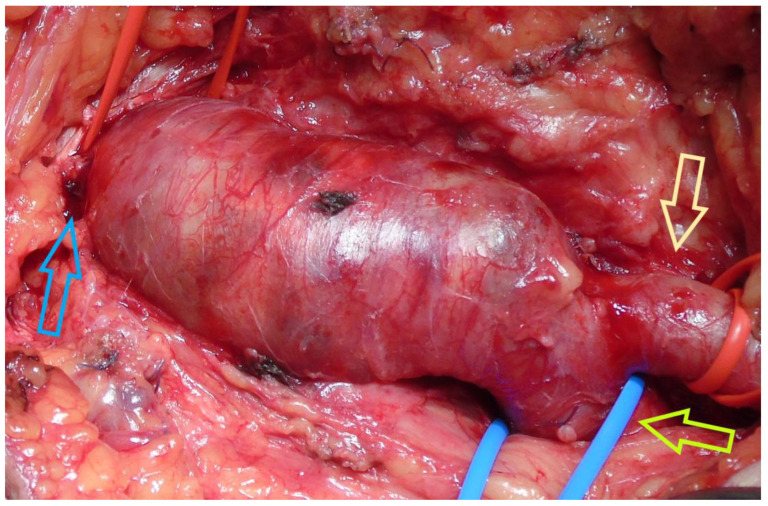
The common femoral artery aneurysm after groin dissection (blue arrow: aneurysm’s neck, light brown arrow: superficial femoral artery, light green arrow: profunda femoris artery).

**Figure 7:**
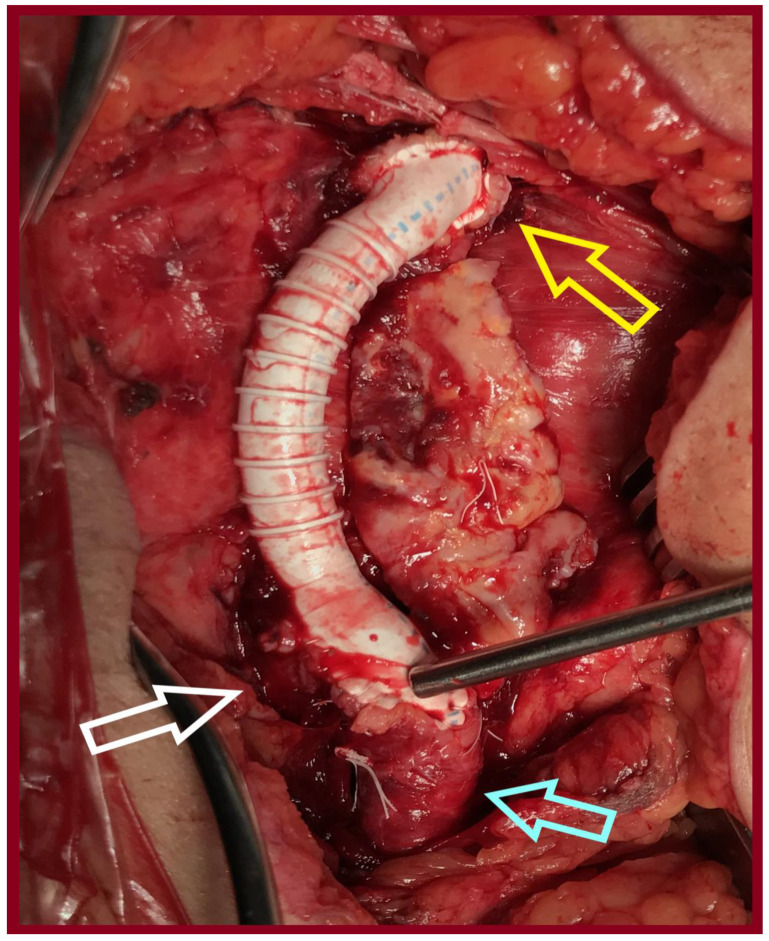
An 8 mm-Ringed PTFE tube graft was interposed between the proximal common femoral artery and the common femoral bifurcation (yellow arrow: proximal anastomosis, light blue arrow: Superficial femoral artery, white arrow: profunda femoris artery).

**Figure 8:**
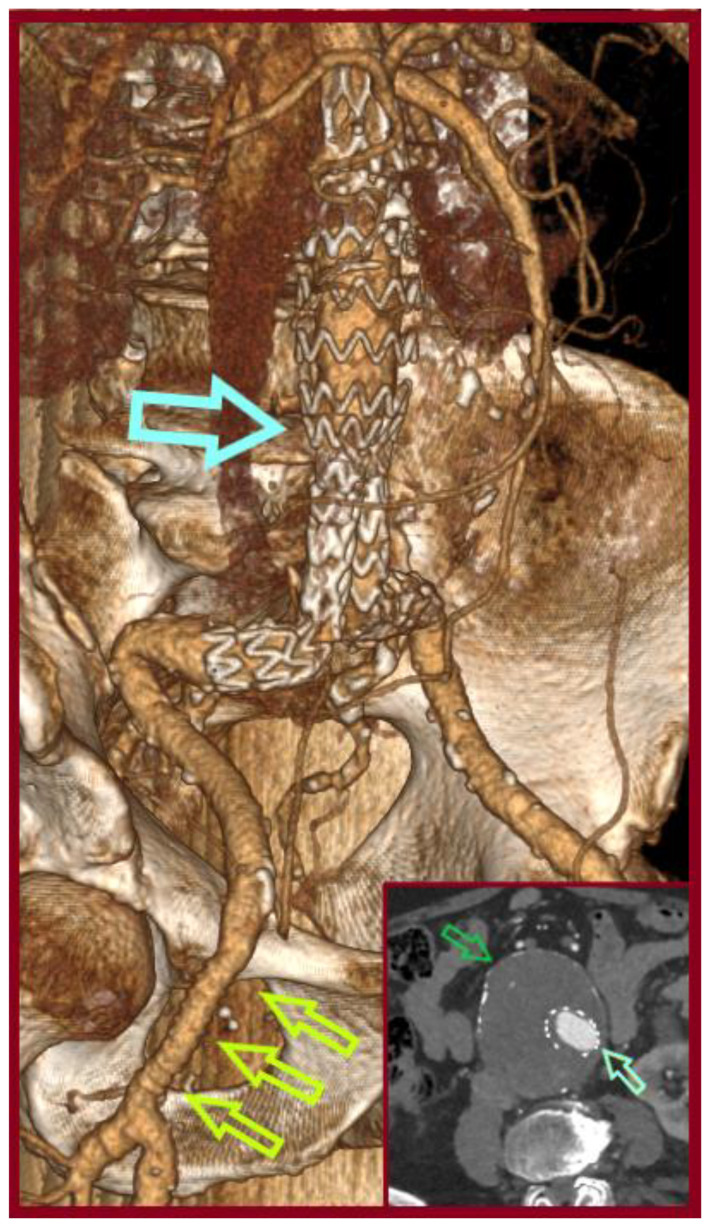
Computed Tomography Angiography (CTA, 3-D Reconstruction) depicting a stable aortic sac without endoleak (green arrow in the incorporated axial view), at 3-years follow-up, (light blue arrow: the endograft, light green arrows: the PTFE graft at the right groin).

A 77-year-old male was transferred from a local hospital due to an intact AAA, 8.3 cm in diameter, diagnosed after a fall attack. A concomitant right CFAA was apparent on CTA, sized 3 cm in diameter, and a right PA sized 1.9 cm. The CFAA was also depicted on a color duplex ultrasound (Figure 5). He was an active smoker, and his past medical history included dyslipidemia, chronic obstructive pulmonary disease, and arterial hypertension. He received atorvastatin 20 mg, o.d., indacaterol maleate 85 mg plus chloroglycopyrronium bromide 43 mg o.d., and amlodipine 5 mg o.d. Operative management included a one-stage approach under general anesthesia. After femoral cutdown, the right CFAA was dissected and looped at the neck and the CFA bifurcation (Figure 6). A standard EVAR was accomplished with a Zenith Flex endograft (Cook Medical, Bloomington, IN, USA). Vascular access was gained through the origin of the SFA. Consequently, a standard CFAA repair was performed with interposition of an 8 mm Ringed e-PTFE graft (W. L. Gore & Associates Inc., Flagstaff, AZ, USA) (Figure 7). The distal anastomosis was completed on the CFA bifurcation. Postoperative outcome was uneventful, and he was discharged on the fourth p.o. day under Clopidogrel. Annual follow-up imaging was normal. He remains in good condition 3 years later (Figure 8). The PA aneurysm remained stable on regular color duplex imaging.

### 3.3. Case 3

**Figure 9:**
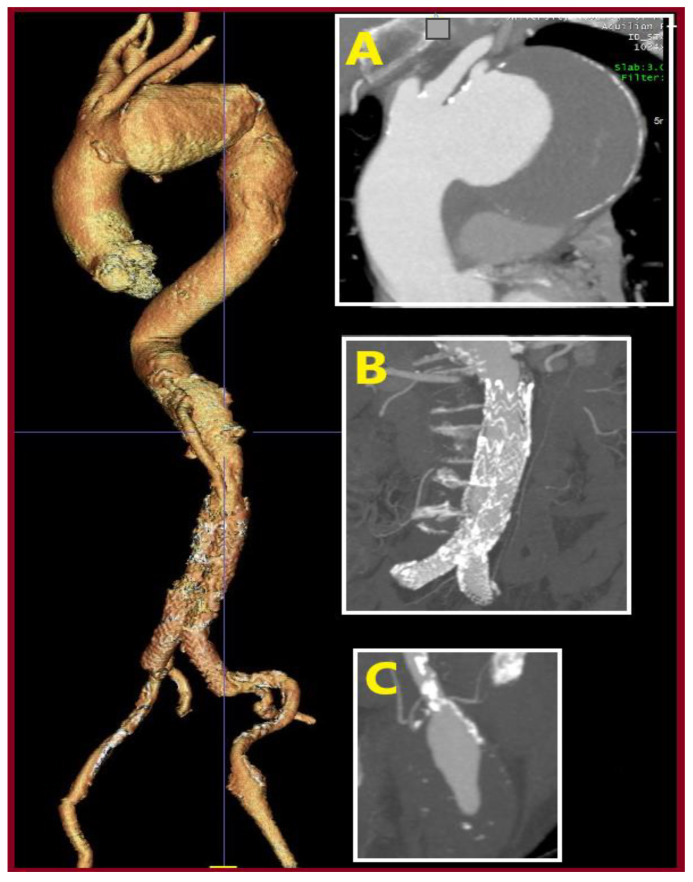
Computed Tomography Angiography (CTA, 3-D Reconstruction) depicting a totally shrunken aortic sac without endoleak, 10 years postoperatively. The graft at the right groin is patent. Unfortunately, two new aneurysms have arisen. (**A**): A newly diagnosed thoracic aortic aneurysm; (**B**): the old endograft; (**C**): a newly diagnosed left common femoral artery aneurysm).

A 62-year-old male was admitted with a symptomatic 5.5 cm AAA and an asymptomatic right CFAA 3.3cm in diameter detected incidentally on CTA. He was an active smoker, and his past medical history included arterial hypertension, dyslipidemia, hyperuricemia, and a coronary angioplasty due to a myocardial infarction, 9 years before admission. He received acetylsalicylic acid 100 o.d., irbesartan 150 mg plus hydrochlorothiazide 12.5 mg o.d., allopurinol 100 mg o.d., sotalol hydrochloride 80 mg b.i.d., and ezetimibe 10 mg plus simvastatin 10 mg o.d. Operative management included a hybrid approach under general anesthesia. After femoral cutdown, the right CFAA was dissected and looped at the neck and the CFA bifurcation. A standard EVAR was accomplished with a Gore Excluder Endoprosthesis (W. L. Gore & Associates Inc., Flagstaff, AZ, USA). The main body component was introduced through the left CFA and the contralateral leg through the origin of the right SFA. Consequently, a standard CFAA repair was performed with interposition of an 8 mm Ringed e-PTFE graft (W. L. Gore & Associates Inc., Flagstaff, AZ, USA). The distal anastomosis was completed on the CFA bifurcation. Postoperative outcome was uneventful, and he was discharged on the third p.o. day. He did not perform follow-up imaging despite his physician’s advice for the following 10 years. Currently, a CTA revealed AAA sac shrinkage, but a new thoracic aortic aneurysm, 9 cm in diameter, and a 4.2 cm-sized left CFAA had emerged (Figure 9). These aneurysms are scheduled for repair.

## 4. Discussion and Literature Review

The normal size of a common femoral artery (CFA) is approximately 1.0 cm in men and 0.8 cm in women, and a 50% fusiform increase in diameter sets the diagnosis of an aneurysm [1]. True CFAAs are rare entities. They account for 2–5% of all peripheral arterial aneurysms. Their incidence varies between 0.3 and 0.5% and is higher among smokers, males, and those with atherosclerosis, increased age, and a positive family history [1,29]. They most likely present in males more than 70 years old [30]. The incidence of CFAAs among FAAs ranges between 57 and 81%, while the incidence of superficial femoral artery aneurysms (SFAAs) and profunda femoris artery aneurysms (PFAAs) ranges between 14 and 26% and 5–17% respectively [31,32,33,34]. Generally, FAAs occur in approximately 5 per 100,000 patients, with 30–66% presenting asymptomatically [22]. Twenty-six per cent of FAAs are bilateral [1]. Cutler and Darling suggested a classification of CFAA into 2 types based on the degree of CFA bifurcation involvement [35]. In type I the aneurysmal disease affects only the CFA (the bifurcation is normal), while in type II, the origin of the PFA is involved [35]. Imaging modalities to assess FAA include CTA, duplex ultrasound, digital subtraction angiography (DSA), and Magnetic Resonance Imaging (MRI) or Magnetic Resonance Angiography (MRA) [31,36]. After diagnosis of a peripheral artery aneurysm, investigation to exclude other thoracic, aortoiliac, femoral, and popliteal aneurysms should be performed [30].

Indication for repair in asymptomatic FAAs is based on a case series of limited size [36]. Repair has been considered clinically indicated for all femoral aneurysms more than 2.5 cm in diameter, but data from 2014 suggested an increased size to a 3.5 cm diameter in isolated FAAs [31]. This results from a lack of adequate knowledge regarding the natural history of FAAs. Based on current available evidence, it seems that smaller aneurysms may be more benign than previously thought, as acute complications are more likely to occur in aneurysms > 3.5 cm or in the presence of intraluminal thrombus [22,25,31,36,37,38]. Consequently, the size threshold for elective repair should be reduced in the presence of intraluminal thrombus [1]. The 2.5 cm diameter threshold was generated in previous decades, when precise vascular imaging with modern equipment was not available and studies included a mixed population of traumatic pseudoaneurysms (e.g., after catheterization), anastomotic pseudoaneurysms, and degenerative aneurysms [31]. In a recent study the mean diameter of operated SFAAs was 5.41 cm (2.7–15.5) [39].

Symptomatic and complicated FAAs warrant repair [30]. Symptomatic patients more often experience a palpable mass, localized pain, or claudication rather than rupture or acute thrombosis [22,40]. Accumulative experience shows that most often they remain silent until life-threatening complications emerge [30]. Main complications are rupture, embolization, thrombosis, and locally compressive symptoms, which may lead to threatening limb ischemia [5,20,22,32,40,41]. Local pain and tenderness may be due to aneurysm expansion (imminent rupture) or nerve compression [28,32]. Venous compression may lead to leg edema or deep venous thrombosis and pulmonary embolism [30,42,43]. CFAA rupture has not occurred so far for diameters < 5 cm [29,30,31]. Large FAAs (>5 cm) have an annual rupture rate as high as 16% [25,40]. Although in older studies, the rates of FAA thrombosis and embolization were 15% in CFA, 45% in profunda femoris artery (PFA), and 26% in SFA, in a newer series, it is only 3%, and the rupture is 4% for all the above arteries [30,31,42,44]. These results were also verified in a recent study of 49 FAAs, where 3 ruptured, 4 thrombosed, and 2 presented with distal embolization [39].

The standard treatment option for CFAAs is open partial aneurysmectomy and interposition grafting. Prosthetic grafts fit better than vein grafts regarding the vessel size and have equivalent or better patency rates than vein grafts in this position [1]. The exact type of reconstruction is based on Cutler and Darling’s classification [36]. Small CFAAs could be treated with excision and end-to-end anastomosis [20,36]. This was performed for the first time by Fagg in 1908 [45]. In specific anatomic circumstances, a part of an axillary-bifemoral bifurcated graft can be used to restore the CFA bifurcation [20,22]. SFAAs might additionally be treated with ligation and bypass or simple ligation in high-risk patients [26,42,43,46,47,48]. Embolization is an alternative treatment for PFAs [34,42,49]. Open surgery is traditionally considered the gold standard for the treatment of FAAs [21,39]. It additionally offers mass decompression compared with endovascular treatment [39]. Five-year survival rate was 77.6% for the 31 operated patients in a series of 35 FAAs reported by Piffaretti et al. [33]. Endovascular treatment has gained popularity over the last years and may become the preferred choice in the future [39]. Reported cases with total endovascular treatment and hybrid (combined open and endovascular) in CFAAs are scarce in the literature [32,49,50,51]. Regarding SFAAs, endovascular treatment is mainly adopted in the medial third of the SFA [39]. Potential complications of endovascular therapy are covered: stent fracture or migration and thrombosis [22,25,52,53]. We must underline that endografting does not relieve the compressive symptoms [32]. Additionally, as more than one-third of FAAs are bilateral, contralateral access for antegrade endograft deployment may be complicated with pseudoaneurysm formation at the puncture site [32].

When more than one aneurysm coexists, the aneurysm with the highest risk for complications is treated first [22,30]. This would be accomplished with staged procedures [22,42,54]. In one report, AAA reconstruction was performed 6 months after CFAA repair [40]. However, ruptured AAA has been reported in patients who underwent initial CFAA repair and deferred aortic surgery [35]. In another recent report, a patient with Loeys-Dietz syndrome type V presented with a true PFAA and a common iliac artery aneurysm (CIAA). One year after a standard EVAR including IAA coiling, open aneurysmectomy with ligation of the PFA was performed [55]. Although the consensus for the treatment of synchronous or metachronous aneurysms is to stage the procedures, a single and simple combined operation, like our approach, would be prudent [22,56]. This approach, if feasible, may lead to decreased medical expenses, decreased hospitalization, and potentially decreased complications [22]. Simultaneous open treatment of CFAA and PA has already been reported [17]. We must bear in mind that similar combined procedures are already performed in nearly 1 in 5 patients in standard EVAR. These patients need CFA patch angioplasty plus endarterectomy or femoro-femoral bypass due to CFA atherosclerosis. Interestingly, these patients had decreased survival in a previous study (79% vs. 58% at 2 years) [57].

Generally, the presence of intraluminal thrombus has been considered as a risk factor for complications like distal embolism and aneurysm thrombosis leading to limb ischemia. This risk is further exacerbated in the context of concurrent EVAR due to manipulations in the access sites, like sheath insertion, external compression, etc. This is true whether the access is percutaneous or via femoral cutdown. Additionally, if someone chooses staged procedures and the access during EVAR is via femoral cutdown, a redo operation to treat the CFAA would be more difficult due to adhesion in the operative field. These concerns justify the treatment of AAA and CFAA in one stage, especially in the presence of intraluminal thrombus [30].

We performed a Scoping review according to PRISMA-ScR criteria. Major medical databases, including PubMed, Scopus, Web of Science, and the Cochrane Database of Systematic Reviews were encountered. The search strategy incorporated the following keywords: “abdominal aortic aneurysm”, “common femoral aneurysm”, “femoral aneurysm”, “endovascular abdominal aortic aneurysm repair”, “endovascular repair”, “open repair” and combinations of them. Retrieved records were subsequently screened for relevance to the topic of the study. A cross-reference was carried out to retrieve relevant studies.

Our literature review (summarized in Table 2) underscores the rarity yet clinical significance of simultaneous endovascular abdominal aortic aneurysm repair and open repair of a common femoral artery aneurysm, since only four case reports and one small case series with two relevant patients (out of six) were retrieved in the published English literature. In four patients, the CFAA was treated with the standard open inlay technique, while the remaining two were treated with less invasive open hybrid techniques. No cases with SFAA or PFAA and simultaneous EVAR were revealed in the literature.

The first case of one-stage repair was reported by Wolthuis AM et al. in 2006. A 74-year-old male with a history of open interposition grafting for a ruptured AAA 10 years previously, presented with a right CIAA and bilateral CFAAs. An aorto-uni-iliac stent graft excluded the right CIAA after right internal iliac artery (IIA) coiling, while an iliac occluder blocked the left common iliac artery (CIA). CFAA repair was achieved with partial (on the right side) and total (on the left side) aneurysm excision and femoro-iliaco-femoral Y-bypass grafting [58].

The second case and third cases were reported by Rancic et al. in 2013 [32]. The patients were males, 79 and 65 years old, with AKAs of 6.5 cm and 6 cm, respectively. Access was gained through limited dissection of the anterior aneurysmal wall. A standard EVAR was initially performed. Afterward, an open and less invasive hybrid repair of the CFAA was performed as described in detail in another part of the discussion section (see below) [32].

The fourth case was reported by Dolapoglu A et al. in 2017 [30]. A 67-year-old male presented with a right palpable CFAA and bilateral CIAAs. Repair of the CFAA was achieved with aneurysmectomy and interposition of a bifurcated graft. Subsequently, stent-grafts were inserted through a temporary conduit on the bifurcated graft, after IIA coiling [30].

The fifth case was reported by Vijayakumar V et al. in 2024 [36]. A 70-year-old male presented with an asymptomatic left CFAA and a known AAA with rapid growth. He underwent a standard EVAR for his AAA. Afterward, an interposition grafting was performed to treat the left CFAA [36].

The sixth case was reported by the Kennedy RE et al. in 2024. A 64-year-old male presented with lower abdominal and left groin pain. An AAA, bilateral CIAAs, and a left CFAA were revealed. A complex EVAR was performed, plus an iliac branch endograft. Access was gained through the neck of the CFAA. Then, a bifurcated interposition grafting was performed subsequent to resection of the CFAA [22].

The case reported by Wolthuis AΜ et al. differed from our patient in six points. First, he had successfully treated a ruptured AAA 10 years previously by open reconstruction. Second, he had a right CIAA (instead of an AAA). Third, the CFAAs were bilateral. Fourth, embolization of the right IIA was performed one day before the operation. Fifth, an aorto-uni-iliac endograft was used (instead of a bifurcated one) and an occlusive device in the left CIA. Sixth, a femorofemoral bifurcated graft was used to revascularize the left axis [58]. In the two cases reported by Rancic et al., access was gained through limited dissection of the anterior aneurysmal wall, and a novel hybrid repair of the CFAA was performed as described elsewhere in this article [32]. The case reported by Dolapoglou et al. differed from our patient in three ways. First, there were bilateral common iliac aneurysms (instead of an AAA). Second, the CFAA was type II and treated with interposition of a bifurcated Dacron graft. Third, the CFAA was treated first in the same procedure. Fourth, an extra conduit was anastomosed on the CFAA graft to give access for stented grafts to treat the CIAAs. No details about the type of these stented-grafts are given [30]. Access in the report by Vijayakumar V et al. was gained percutaneously [36]. The contralateral limb was inserted through the left side, where the CFAA was located, as it had a smaller profile. It is not reported if the sheath was introduced proximally or distally to the aneurysm. After completion of the aortic endograft placement, open repair of the CFAA was accomplished [3]. Access in the report by Kennedy et al. was gained through the proximal CFAA sac and not via the SFA as in our case [22]. Insertion through SFA, as we performed in our patients, is an alternative to CFA with lower complication rates [59,60]. Additionally, an iliac branch endoprosthesis (IBE) was used on the right side due to short and aneurysmal CIA. A bifurcated Dacron instead of a straight PTFE graft (as in our case) was used to repair the CFAA, as the femoral orifices had moved apart due to CFAA extension to the bifurcation. Despite the use of an IBE and a bifurcated femoral graft, no extension of hospitalization was required [22].

Undoubtedly, the benefits of a combined repair are focused on the decreased number of hospitalizations, reduced medical costs, and increased patient satisfaction. In a combined approach, potential AAA rupture is avoided in the meantime, compared with single repair of a CFAA and deferred aortic surgery [35]. If the CFAA inhabits intraluminal thrombus, a single EVAR, regardless of access type, would potentially provoke the CFAA thrombosis of distal thromboembolism, both of which are combined with limb-threatened complications [30]. Surgical field adhesions in a redo operation are another issue. These concerns justify the combined treatment of AAA and CFAA in one stage.

## 5. Conclusions

Combining EVAR and open repair of CFAAs by interposition grafting in one stage is a simple procedure that led to satisfactory outcomes in our patient population. Analogous cases have been reported in the literature. Perhaps, these one-stage procedures reduce the number of hospitalizations, which results in reduced medical costs and increased patient satisfaction. Moreover, there is evidence of surgeons’ satisfaction due to good results achieved with a one-stage approach in a total of six reported cases. Our experience enriches the current literature with three additional cases. Consequently, we recommend combined EVAR and CFAA open repair when the indication for treatment is present.

## Figures and Tables

**Table 1:** Demographic, imaging, and operative data of our patients.

Patient ID	Age/Sex	AAA/Size (cm)	CIAA Coexistence	CFAA Side/Size (cm)	AAA Treatment	CFAA Treatment	Order
Patient 1	76/M	Yes/5.5	No	R/2.5	Standard EVAR	Straight interposition grafting	First the AAA
Patient 2	77/M	Yes/8.3	No	R/3	Standard EVAR	Straight interposition grafting	First the AAA
Patient 3	62/M	Yes/5.5	No	R/3.3	Standard EVAR	Straight interposition grafting	First the AAA

Abbreviations: AAA: Abdominal aortic aneurysm, CIAA: Common iliac artery aneurysm, CFAA: Common femoral artery aneurysm, M: Male, R: Right, EVAR: Endovascular abdominal aortic aneurysm repair.

**Table 2:** All reported cases of less invasive open (or hybrid) repair for common femoral artery aneurysms with simultaneous EVAR.

Pt ID	Author/Ref	Year	Age/Sex	AAA/Size (cm)	CIAA/Side/Size (cm)	CFAA Side/Size (cm)	AAA Treatment	CIAA Treatment	CFAA Treatment	Order
1	Wolthuis AM et al./[58]	Surgeon. 2006	74/M	No	Yes/R/3.2	Bilate-Ral/R: 6 & L: 5	Open interposition grafting for rupture, In the past	AUI (RIAA coiling, LCIA occluder)	Femoro-iliaco-femoral Y-bypass grafting	First the CIAA
2	Rancic Z et al./[32]	EJVES 2013	79/M	Yes/6.5	No	NR/6.5	Standard EVAR	-	Hybrid	First the AAA
3	Rancic Z et al./[32]	EJVES 2013	65/M	Yes/6	No	NR/3.7	Standard EVAR	-	Hybrid	First the AAA
4	Dolapoglu A et al./[30]	SAGE Open Med Case Rep 2017	67/M	No	Yes/Bilate-ral/NR	R/NR	-	Stented grafts–IIAs coiling (Conduit was used for access)	Bi-furcated inter-position grafting	First the CFAA
5	Vijayakumar V et al./[36]	BMJ Case Rep 2024	70/M	Yes/5.4	No	L/2.4	Standard EVAR	-	Straight inter- position grafting	First the AAA percutaneously
6	Kennedy RE et al./[22]	Vascular 2024	65/M	Yes/4.4	Yes/Bilate-Ral/R: 2.8 & L: 2.7	L/4.5	Complex EVAR ^1^	Complex EVAR ^1^	Bi-furcated inter-position grafting	First the AAA & CIAAs

Abbreviations: AAA: Abdominal aortic aneurysm, CIAA: Common iliac artery aneurysm, CFAA: Common femoral artery aneurysm, AUI: Aorto-uni-iliac stent-graft, RIIAA: Right internal iliac artery aneurysm, LCIA: Left common iliac artery, RCFAA: Right common femoral artery aneurysm, LCFAA: Left common femoral artery aneurysm, IIA: Internal iliac artery, NA: Not reported, M: Male, R: Right, L: Left, EVAR: Endovascular abdominal aortic aneurysm repair, ^1^: EVAR plus Iliac Branch Endoprosthesis.

## Data Availability

Research data are stored in an institutional repository and will be shared upon reasonable request to the corresponding author.
